# Reappraising the evolutionary history of the largest known gecko, the presumably extinct *Hoplodactylus delcourti*, via high-throughput sequencing of archival DNA

**DOI:** 10.1038/s41598-023-35210-8

**Published:** 2023-06-19

**Authors:** Matthew P. Heinicke, Stuart V. Nielsen, Aaron M. Bauer, Ryan Kelly, Anthony J. Geneva, Juan D. Daza, Shannon E. Keating, Tony Gamble

**Affiliations:** 1 University of Michigan–Dearborn, Dearborn, MI USA; 2 Louisiana State University Shreveport, Shreveport, LA USA; 3 Florida Museum of Natural History, University of Florida, Gainesville, FL USA; 4 Villanova University, Villanova, PA USA; 5 Rutgers University–Camden, Camden, NJ USA; 6 Sam Houston State University, Huntsville, TX USA; 7 Marquette University, Milwaukee, WI USA; 8 The Bell Museum of Natural History, University of Minnesota, Saint Paul, MN USA; 9 Milwaukee Public Museum, Milwaukee, WI USA

**Keywords:** Evolution, Phylogenetics, Taxonomy

## Abstract

*Hoplodactylus delcourti* is a presumably extinct species of diplodactylid gecko known only from a single specimen of unknown provenance. It is by far the largest known gekkotan, approximately 50% longer than the next largest-known species. It has been considered a member of the New Zealand endemic genus *Hoplodactylus* based on external morphological features including shared toe pad structure. We obtained DNA from a bone sample of the only known specimen to generate high-throughput sequence data suitable for phylogenetic analysis of its evolutionary history. Complementary sequence data were obtained from a broad sample of diplodactylid geckos. Our results indicate that the species is not most closely related to extant *Hoplodactylus* or any other New Zealand gecko. Instead, it is a member of a clade whose living species are endemic to New Caledonia. Phylogenetic comparative analyses indicate that the New Caledonian diplodactylid clade has evolved significantly more disparate body sizes than either the Australian or New Zealand clades. Toe pad structure has changed repeatedly across diplodactylids, including multiple times in the New Caledonia clade, partially explaining the convergence in form between *H. delcourti* and New Zealand *Hoplodactylus*. Based on the phylogenetic results, we place *H. delcourti* in a new genus.

## Introduction

The more than 2150 described geckos and flap-footed lizards (infraorder Gekkota) constitute one of the most species-rich assemblages of terrestrial vertebrates. This species richness extends to all parts of the world with warm climates^1^ and is defined in part by relatively morphologically conservative regional radiations with high degrees of local endemism, such as African *Pachydactylus*, Asian *Cyrtodactylus*, Australian *Gehyra*, and Neotropical *Sphaerodactylus*^2–5^. Some regional radiations of geckos exhibit notable diversity in body form and life history in addition to high species richness. The family Diplodactylidae is one example. The ~ 190 species of diplodactylid geckos are restricted to Australia, New Caledonia, and New Zealand, and form a substantial proportion of the reptile fauna in each of these regions^1,6^. New Caledonian and New Zealand taxa each constitute separate monophyletic radiations^7,8^. Diplodactylids occur in habitats ranging from desert to grassland to forest, may be terrestrial, rock-dwelling, or arboreal, may be nocturnal or diurnal, and vary greatly in phenotypic characteristics such as body size, tail shape, pupil type, and possession and type of toepads, interdigital webbing, and skin flaps^9–11^. One morphologically extreme example of the diplodactylid radiation is the largest extant gecko, the New Caledonian *Rhacodactylus leachianus* (maximum snout-vent length 255 mm^9^).

**Figure 1:**
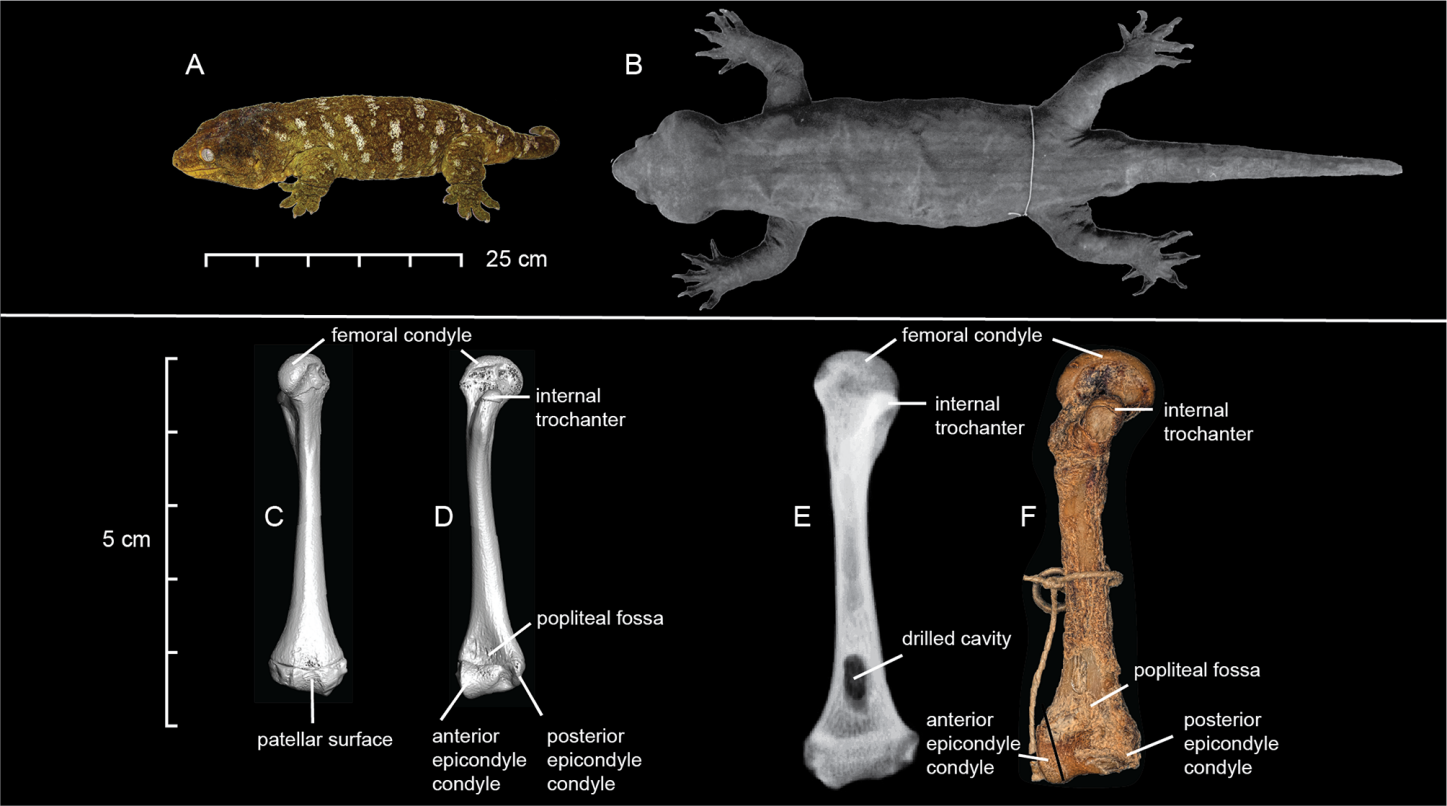
*Hoplodactylus delcourti* compared to the largest extant gecko, *Rhacodactylus leachianus*. (**a**), *R. leachianus* (captive), and (**b**), *H. delcourti* (MMNH 1985-38), external morphology. (**c–d**), High resolution CT of *R. leachianus* (MCZ Herp R-15967). (**e**), X-ray of the left femur of *Hoplodactylus delcourtii* (MMNH 1985-38), and (**f**), digital photograph of the specimen in posterior view. (**a**, **b**), (**c**–**f**) shown to scale. The femurs of *H. delcourti* and *R. leachianus* are very similar in overall morphology, with differences reflecting an allometric relationship. The femur of *R. leachianus* is 77% the length of the femur *of H. delcourti*, and the width is 57%. Although the femoral condyle seems more prominent in *H. delcourti*, this is due to the preservation of the articular cartilage. In *R. leachianus*, the neck of the internal trochanter is narrower, the shaft of the body is more curved and the polipteal fossa is deeper. Images of MCZ Herp R-15967 are courtesy of the Museum of Comparative Zoology at Harvard University.

Even larger is the presumably extinct species *Hoplodactylus delcourti*. This species was described from a single specimen that was rediscovered among the collections of the Musêe d’Histoire Naturelle de Marseille, France^12^. Its provenance and date of collection were uncertain, but it had likely been part of the collections since at least 1870, and on the basis of style of preservation, the 1830s. At 370 mm snout-vent length (SVL), the specimen is about 50% longer than the largest recorded *Rhacodactylus leachianus* (Fig. 1). Its preservation is unusual for reptiles, which are typically preserved whole in spirits. Instead, the specimen was eviscerated, dried and mounted, lacking the internal organs and most of the axial skeleton, but possessing the skull and appendicular skeleton (Fig. 1). This state of preservation hinders assessment of some morphological characters, and makes dating unreliable. There was sufficient morphological evidence for Bauer and Russell^12^ to conclude that it belonged to the family Diplodactylidae, likely a member of the New Zealand genus *Hoplodactylus*. The authors also considered a New Caledonian origin, but ultimately placed the specimen in *Hoplodactylus* based on color pattern and digital morphology. However, a subsequent phylogenetic analysis of 107 morphological traits—of which 57 were obtainable from the *H. delcourti* specimen—placed it in an unresolved polytomy among both New Zealand and New Caledonian diplodactylids^13^.

The specimen also resembles a lizard known from Māori folklore. Bauer and Russell^14^ reviewed historical literature reporting Māori folk knowledge of large reptiles. One of these species known to the Māori was the *kaweau* or *kawekaweau*, a ‘large forest lizard,’ reported from several areas of New Zealand’s North Island, and the anecdotal description matches the size and color of the preserved *Hoplodactylus delcourti* specimen. However, extensive fossil and subfossil material has failed to document the presence of *H. delcourti* in New Zealand, though other extinct geckos related to the largest extant New Zealand species, *H. duvaucelii*, have been recorded^15–17^.

Given the uncertainty surrounding the geographic origin and evolutionary relationships of *Hoplodactylus delcourti*, we sought to obtain sequence data to test its phylogenetic affinities. Reliable phylogenetic placement of *H. delcourti* is also necessary to evaluate broader evolutionary patterns across Diplodactylidae in a regional context. Furthermore, reliable phylogenetic placement of the largest gecko species is especially crucial for assessing patterns of body size evolution, and a secondary goal of our study is to determine whether this giant gecko belongs to a lineage exhibiting anomalously rapid body size evolution. It also possesses additional uncommon morphological characters, including rectangular digital pads and interdigital webbing. As digital pad shape and webbing exhibit extensive variation across Diplodactylidae the evolutionary context of these traits in *H. delcourti* is not resolved.

We successfully obtained DNA from arthroscopically removed bone and generated sequence data, including a complete mitogenome, from the only known specimen of *Hoplodactylus delcourti*. We use these data to perform a set of phylogenetic analyses on multi-locus and mitogenomic data sets of up to 169 species incorporating near-complete taxon sampling across Diplodactylidae. We use the results of these analyses to assess the taxonomy of *H. delcourti* and perform phylogenetic comparative analyses to determine patterns of biogeographic history and phenotypic evolution of three traits: body size, toepads, and skin folds and webbing. In combination, these analyses permit comparisons between the Australian diplodactylids and the insular radiations of New Caledonia and New Zealand, which have evolved in the absence of other geckos. We are particularly interested in evaluating whether the massive size of *H. delcourti* is accompanied by a shift in the rate of body size evolution in its recent ancestors.

## Results

### Phylogeny

**Figure 2:**
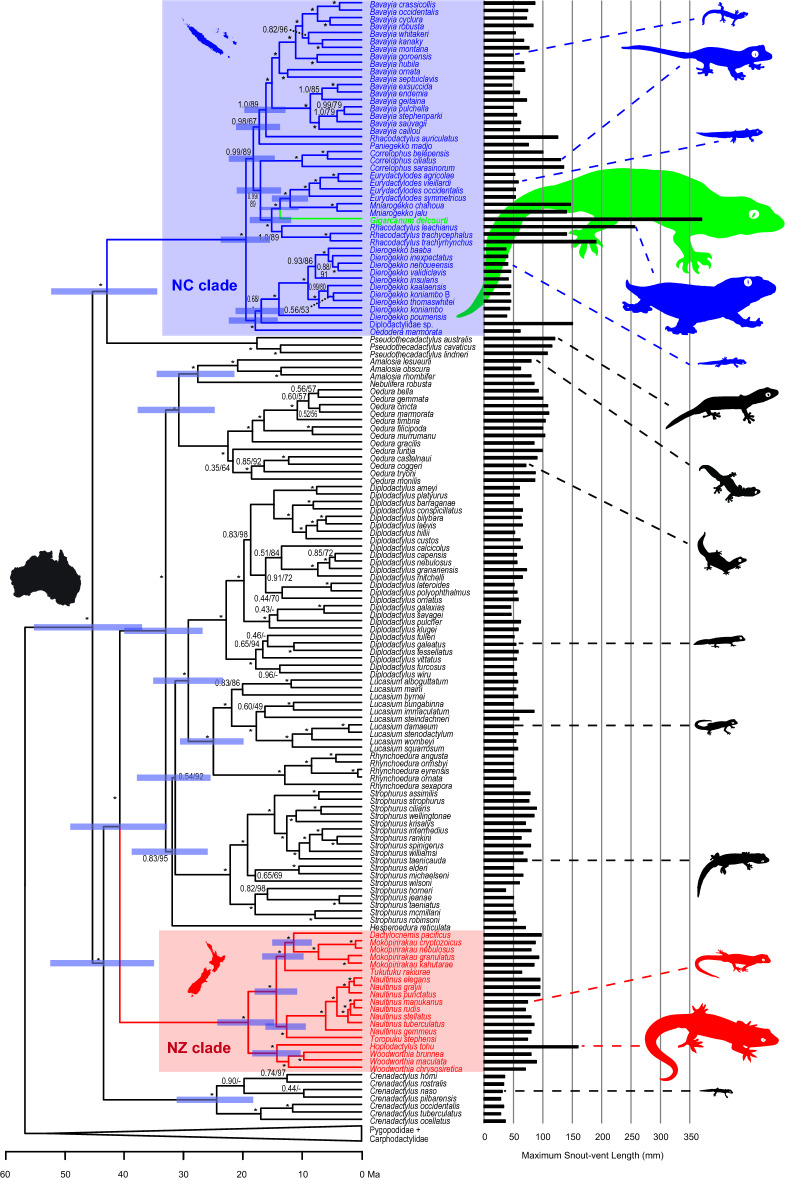
Time-calibrated maximum clade credibility phylogeny of Diplodactylidae based on the 169 taxon multilocus data set. 95% highest posterior density intervals of divergence times and branch support values (Bayesian posterior probabilities/ML bootstrap) are given at nodes. Asterisks denote nodes with maximum branch support. Species maximum body sizes (SVL) and geographic ranges are indicated.

The multi-locus DNA sequence alignment includes 11,806 positions across 15 genes and was analyzed using three sampling strategies: using all available data for 169 species, using all genes for 30 species to minimize missing data, and using 10,126 positions and 13 genes for 143 species to analyze nuclear genes alone. The mitogenomic alignment includes 16,375 positions for 39 species. The assembled mitogenome of *Hoplodactylus delcourti* contains 13 protein-coding genes, 22 tRNA genes, two rRNA genes, and the control region, conforming to the typical vertebrate gene order. All four data sets recover tree topologies that are congruent at key nodes (Supplementary Figs. S1–S5 online), so patterns described herein refer to the 169 species phylogeny (Fig. 2). Intergeneric patterns mirror relationships observed in published analyses (e.g.,^7,8,18,19^). Diplodactylidae is monophyletic with maximum nodal support (ML Bootstrap = 100, Bayesian PP = 1.0). The deepest divergence within Diplodactylidae is between two strongly supported clades, one including most Australian and New Zealand taxa on one hand (support values 100/1.0), and the other including *Pseudothecadactylus* + New Caledonian taxa (support values 95/0.99). Extant New Zealand taxa form a monophyletic group (support values 100/1.0), as do extant New Caledonian taxa (support values 100/1.0). However, *H. delcourti* is not recovered as most closely related to the extant *Hoplodactylus* species *H. tohu*, or as a member of the New Zealand clade at all. Instead, it is embedded in the New Caledonia clade. Evolutionary relationships within the New Caledonia clade are not well resolved across the four data sets, but in all cases *H. delcourti* is embedded in this clade with maximum nodal support and is most closely related to some combination of *Eurydactylodes*, *Mniarogekko*, and/or *Rhacodactylus*.

Topological tests strongly reject the monophyly of *Hoplodactylus* (including *H. delcourti*) and reject an association of *H. delcourti* with the New Zealand clade. Using the 169 species data set, the ML phylogeny has a much higher likelihood than trees constrained to enforce monophyly of *Hoplodactylus* (∆logL = 732.95) or place *H. delcourti* with the New Zealand clade (∆logL = 538.55). Both SH (*p* = 0) and AU tests (*p* = 1.56 × 10^−6^) reject the null hypothesis of equivalent likelihoods, indicating topologies that place *H. delcourti* with the New Zealand clade are significantly worse than those placing it within the New Caledonia clade. Topological tests performed using constrained trees with the 30 species data set likewise reject monophyly of a New Zealand clade that includes *H. delcourti* (SH test *p* = 0; AU test *p* = 8.16 × 10^−40^). Because our phylogenetic analyses recover *H. delcourti* as neither closely related to *H. tohu* nor nested within any other diplodactylid genus, we erect a new genus to contain it herein.

### Systematics

**Family Diplodactylidae Underwood, 1954**

***Gigarcanum*** **gen. nov.**

urn:lsid:zoobank.org:pub:509E331A-0803-4170-91D2-42FB8F5D672D

*Type species*. *Hoplodactylus delcourti* Bauer and Russell, 1986^12^

*Etymology*. The name is a combination of two words: the Latin adjective *gigas*, meaning giant and taken from the Ancient Greek **Γίγᾱς**, and the Latin noun *arcanum*, meaning secret or mystery. The combination refers to the size of the type species and the unknown provenance of the only known specimen. The gender is neuter.

*Definition*. A genus of diplodactylid gecko distinguished from all other geckos by the following combination of characters: size massive (SVL to at least 370 mm), approximately 50% longer and several times heavier than the next largest known gecko. Body robust, with cylindrical, tapering, weakly annulated tail. Digits clawed, weakly to moderately webbed between digits I–IV. Digital pads broad, rectangular, with 25–27 lamellae under digit IV of pes. Scalation granular, precloacal pores continuous with femoral pores on under surface of thighs, precloacal spurs present. Skull massive, length approximately 20% of SVL, not co-ossified to overlaying skin. Nasals paired, frontal single, parietal fused. Cloacal bones present. Phalangeal formulae 2-3-4-5-3/2-3-4-5-4 (manus/pes); paraphalangeal elements absent.

*Content*. *Gigarcanum delcourti* comb. nov.

### Comparative analyses

Divergence time estimates within Diplodactylidae broadly overlap estimates in other studies (Fig. 2;^7,8,19–21^). Crown Diplodactylidae diversified beginning in the Eocene epoch, approximately 45 (95% HPD: 54–38) million years ago (Ma). The New Caledonia clade (including *Gigarcanum delcourti*) diverged from *Pseudothecadactylus* soon thereafter, 43 (51–35) Ma. Crown divergences in the New Caledonia clade began 20 (24–16) Ma, and the divergence of *G. delcourti* from extant New Caledonian geckos took place 14 (17–11) Ma. The New Zealand clade is comparable in age to the New Caledonia clade: it diverged from its Australian relatives 40 (48–32) Ma, and diversified beginning 19 (24–14) Ma. Ancestral reconstruction of geographic distributions supports an Australian origin for Diplodactylidae with an average of 2.0 transitions across the samples, corresponding to the colonizations of New Caledonia and New Zealand, respectively (Fig. 2). The most recent common ancestor of *G. delcourti* and its closest extant relatives is unambiguously reconstructed to have been distributed in New Caledonia (posterior probability = 1.0).

**Figure 3:**
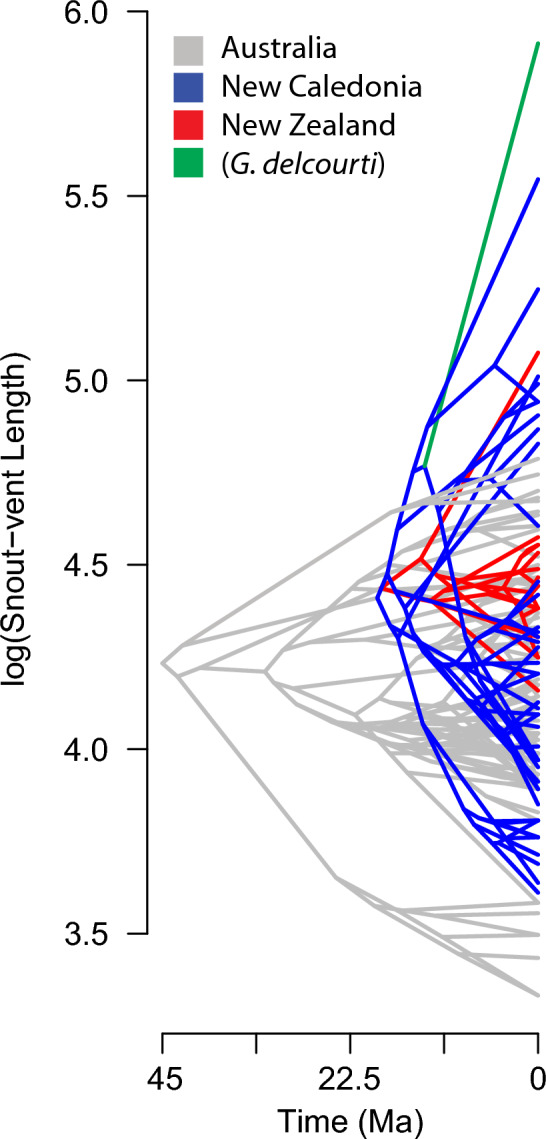
Phenogram depicting evolution of disparity in body size of New Caledonia clade compared to other diplodactylids.

Censored rate test results (Fig. 3; Table 1) indicate variation in the rate of body size evolution (σ^2^) in Diplodactylidae. The New Caledonia clade has a significantly higher rate than both Diplodactylidae as a whole (likelihood ratio: 14.1725, *p* = 2 × 10^−4^) and the New Zealand clade (likelihood ratio: 7.8682, *p* = 0.005). Exclusion of *G. delcourti* in these comparisons does not significantly alter inferred patterns of body size evolution in Diplodactylidae, beyond marginally decreasing rate estimates within the New Caledonia clade; nor does applying the maximum recorded SVL of *Hoplodactylus tohu* (116 mm^22^) in place of *Hoplodactylus duvaucelli* (160 mm). The Bayesian rate shift analysis supports the same inference, with the branch leading to the New Caledonia clade identified as the most likely location of a rate shift in Diplodactylidae in 80% of the posterior sample; the branch leading specifically to *G. delcourti* was not identified as the shift point in any posterior samples.

**Table 1:** Results of censored rate tests comparing common-rate versus multi-rate models of body size evolution in select pairs of diplodactylid clades.

Common-rate model	Multi-rate model		Model comparison		
σ^2^ (± SE)	σ^2^_1_ (± SE)	σ^2^_2_ (± SE)	∆log(L)	likelihood ratio	*p*-value
*NC clade versus Diplodactylidae*					
0.0058 ± 0.0006	0.0106 ± 0.0023	0.0045 ± 0.0005	7.0863	14.1725	0.0002
*NZ clade versus Diplodactylidae*					
0.0043 ± 0.0005	0.0033 ± 0.0011	0.0045 ± 0.0005	0.463	0.9258	0.336
*NC clade versus NZ clade*					
0.0083 ± 0.0015	0.0106 ± 0.0023	0.0033 ± 0.0011	3.9341	7.8682	0.005
*NC clade (G. delcouri pruned) versus Diplodactylidae (G. delcourti pruned)*					
0.0047 ± 0.0005	0.0082 ± 0.0018	0.0037 ± 0.0004	5.6996	11.3992	0.0007
*NC clade (G. delcouri pruned) versus NZ clade*					
0.0066 ± 0.0012	0.0082 ± 0.0018	0.0033 ± 0.0011	2.5312	5.0625	0.0244
*Diplodactylidae versus Diplodactylidae (G. delcorti pruned)*					
0.0041 ± 0.0003	0.0045 ± 0.0005	0.0037 ± 0.0004	0.6022	1.2042	0.2725
*NC clade versus NC clade (G. delcorti pruned)*					
0.0092 ± 0.0014	0.0104 ± 0.0022	0.008 ± 0.0017	0.3638	0.7252	0.3626

“NC clade” includes all New Caledonian taxa plus *Gigarcanum delcourti*. “NZ clade” includes all New Zealand taxa.

**Figure 4:**
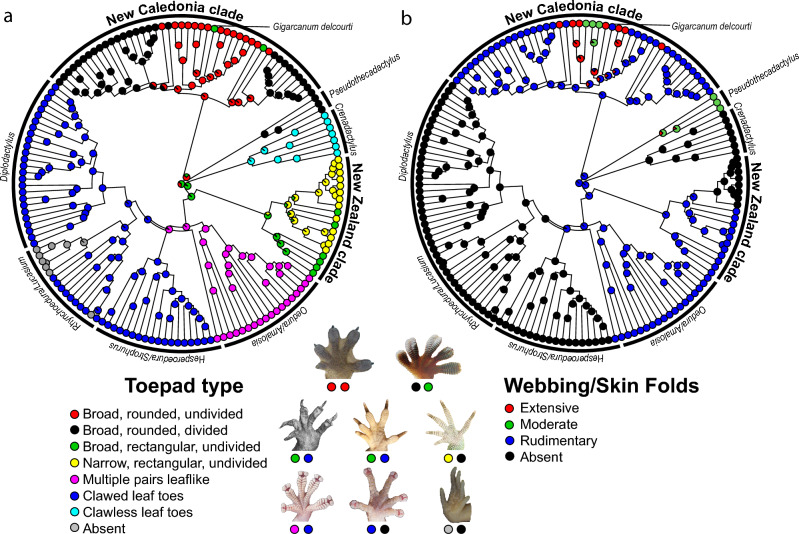
Stochastic character maps of (**a**), toe pad type, and (**b**), possession of webbing and skin flaps in Diplodactylidae. Estimates are based on 1000 iterations using the MCC tree. Photos depicting representative variation are shown for reference. Credits: Tony Gamble (*Rhacodactylus leachianus*, top left), Bjørn Christian Tørrissen, (*Pseudothecadactylus lindneri*, top right, used under CC BY-SA 3.0), Aaron Bauer (*Gigarcanum delcourti*, center left), Dylan Van Winkel (*Hoplodactylus duvaucelii*, center; *Naultinus tuberculatus*, center right), Stephen Zozaya (*Oedura murrumanu*, bottom left, *Diplodactylus nebulosus*, bottom center), Peter Uetz (*Rhyncoedura sexapora* WAM R103413, bottom right).

Ancestral reconstructions of toepad form, extent of skinfolds and webbing show that these traits are labile across Diplodactylidae (Fig. 4). An average of 17.5 shifts in toepad morphology are inferred across the tree, including 5.0 shifts to the broad, rectangular, undivided pad type possessed by *G. delcourti*. Shifts to this toepad type occurred independently in the New Caledonia and New Zealand clades and are absent from Australian lineages. The trait is inferred to have evolved from either a broad, rounded undivided pad type or a narrow, rectangular undivided pad type. The most recent common ancestor of *G. delcourti* and its extant relatives is reconstructed to have possessed broad, rounded, undivided pads (posterior probability = 1.0). Skin folds and webbing are reconstructed to have shifted an average of 15.0 times in Diplodactylidae, including 6.7 shifts to extensive webbing. These shifts are limited to larger-bodied taxa in the New Caledonia clade plus the related Australian genus *Pseudothecadactylus*. The most recent common ancestor of *G. delcourti* and its extant relatives is reconstructed to have possessed rudimentary interdigital webbing, suggesting no change along the branch leading to this taxon (posterior probability = 0.78).

## Discussion

Our results reiterate the potential of osteological samples, and preserved museum specimens in general, as sources of DNA suitable for testing evolutionary hypotheses in recently extinct reptiles (e.g.^23–25^). Thus far, ours and most other studies have focused on larger-bodied taxa. However, the existence of large assemblages of disarticulated bone samples^26,27^ hints at the potential for many more discoveries in this subfield, especially in insular systems where such data alone or guided by analyses of phylogenetic and geographic patterns may permit recognition of otherwise unrecorded anthropogenic extinctions (e.g. *Hoplodactylus*, *Lepidodactylus*^17,28^). In the case of our study, the genetic data force a wholesale reevaluation of the evolutionary context of the largest known gecko.

The biogeographic analysis indicates that *Gigarcanum delcourti* minimally had an ancestor that inhabited New Caledonia, and a most parsimonious explanation would place it in New Caledonia as well. Long-distance dispersal events in Diplodactylidae appear to be much rarer than in gekkonoid geckos, where there are numerous examples of trans-oceanic dispersals (e.g.^29–31^). This difference may stem in part from reproductive differences. Gekkonoid geckos have hard-shelled adhesive eggs that can be relatively impervious to saltwater^32^; thus, eggs deposited in logs or other potential flotsam are viable means of dispersal. In contrast, diplodactylids, presumably including *G. delcourti* and its recent ancestors, either have parchment-shelled eggs or are viviparous. Based on the limited number of examples of long-distance dispersal among diplodactylids we conclude that *G. delcourti* is most likely to have occurred in New Caledonia. However, without direct physical evidence tying the single known specimen to a locality, or the discovery of additional remains (subfossil or otherwise), we cannot rule out the possibility that the species instead inhabited another landmass in the southwest Pacific such as the Loyalty Islands or New Zealand, though colonization of New Zealand would require a successful dispersal and adaptation to a cooler climate. Thus far, detailed investigations of the subfossil herpetofaunal assemblages of New Zealand, New Caledonia, and the Loyalty Islands have not yielded gekkotan remains unambiguously referable to *G. delcourti*^15,26,27,33,34^. One subfossil bone from New Zealand has been interpreted as a gecko cloacal bone of a size consistent with *G. delcourti*^14^, but alternate interpretations have also been proposed^15^. Additional field or historical work, or alternative laboratory approaches such as trace geochemical or palynological analysis, are still needed to clarify the geographic origin of *G. delcourti* and determine whether another species is the best candidate for the identity of the *kawekaweau* of New Zealand. To date, no extant or extinct reptiles known from New Zealand match descriptions of the body size and color of the *kawekaweau* more closely than *G. delcourti*^14^.

Bauer and Russell^12,14^ suggested some aspects of the biology of *Gigarcanum delcourti*, but these were predicated on its close relationship to New Zealand diplodactylids. For example, they speculated^14^ that it may have occupied the crowns of kauri trees. Combined with the inaccessibility of this habitat, kauri forests were shrinking even before the arrival of humans in New Zealand, providing a plausible reason for the fact that only a single specimen is known, although its style of preparation suggests it was collected before terrestrial habitats in either New Caledonia or New Zealand had been heavily impacted by European settlement. Regardless, the presence of broad toepads and well-developed claws suggests it was a climbing species, likely on trees rather than rocks. It was almost certainly nocturnal, like all of its New Caledonian relatives as well as New Zealand diplodactylids exclusive of the distinctive green geckos of the genus *Nautinus*. Its dentition is not exceptional, suggesting a generalized arthropod diet, but it seems likely, given its size, that this would have been supplemented by vertebrate prey (other lizards, nestling birds) and seasonally by fruit, as has been documented in New Caledonian giant geckos^9,35^. *Gigarcanum*’s parity mode is unknowable given the preservation method of the only known specimen. Bauer and Russell^12,14^ assumed it to be viviparous, in line with all New Zealand diplodactylids, but viviparity is also known for two species of *Rhacodactylus*^35^ and cannot be excluded on the basis of its inclusion in the New Caledonian lineage. Whether oviparous or viviparous, a clutch/litter size of two is almost certain as this is fixed in both insular lineages of diplodactylids, regardless of size. In either case the eggs or neonates would likely have been vulnerable to *Rattus* predation^36^. Whatever its biological attributes it is clear that despite its huge size, *G. delcourti* was extinct or exceedingly rare by the time of European colonization of the South West Pacific and that its lifestyle and habitat left it largely or entirely unknown to the pre-European settlers of the region, the brief description of the Maori *kawekauweau*, if indeed referring to this species, providing the only evidence besides the unique specimen that this magnificent lizard ever existed.

Our results place the evolution of gigantism in *Gigarcanum delcourti* within the context of rapidly evolving disparity in body size across the New Caledonia clade of diplodactylids. Thus, although *G. delcourti* is uniquely massive, its evolutionary attainment of large size is not uniquely rapid in comparison to its close relatives. An elevated rate of body size evolution is unique to the New Caledonia clade, as we do not infer the New Zealand clade to have a significantly different rate compared to Diplodactylidae as a whole. This is true even though the size attained by New Zealand *Hoplodactylus* species is similar to some giant members of the New Caledonia clade. This contrasts with the results of Garcia-Porta and Ord^37^, who identified an elevated rate in the New Zealand clade. However, their analyses differed from ours in specifically testing against Australian taxa rather than the family as a whole. Another approach, mean disparity index, employed by Skipwith and Oliver^38^ corroborates our results here in identifying significantly elevated body size disparity in the New Caledonia clade but not the New Zealand clade. These authors did find some evidence that a subset of New Zealand taxa, *Hoplodactylus* + *Woodworthia*, exhibits elevated rates of body size evolution in addition to the New Caledonian taxa.

Of the ~ 18 inferred shifts in toepad morphology across Diplodactylidae, we infer five to have taken place in the New Caledonia clade, one of which was along the branch leading to *Gigarcanum delcourti*. Approximately three shifts have taken place in the New Zealand clade, and the remaining ten among Australian taxa. Regional variation in the number of shifts corresponds with the amount of time that each landmass has been occupied by diplodactylids. Most shifts are between relatively similar pad types, and some shifts have taken place repeatedly. For example, within the New Caledonia clade both *G. delcourti* and a still-undescribed, large-bodied species evolved *Hoplodactylus*-like broad, undivided rectangular pads from ancestors that possessed broad, undivided rounded pads (Bauer et al*.* in prep). Thus, although there are repeated changes in form, individual transitions tend to be relatively limited in scope, in line with observations that the gekkotan adhesion system consists of a suite of structures that have separate evolutionary trajectories such that not all features would be expected to change at a single point in a tree^39^.

We infer the rudimentary digital webbing of *Gigarcanum delcourti* to be a retained ancestral state, and variation in morphology of skin folds and webbing is especially pronounced in the wider New Caledonia clade. Species possessing the most extensive webbing and skin folds, most formerly included in the genus *Rhacodactylus*, are not each other’s closest relatives^35^. Instead, approximately eight transitions to more extensive webbing and skin folds are inferred from an ancestral state of rudimentary webbing in the New Caledonian diplodactylids + *Pseudothecadactylus*, compared to none across the remainder of Diplodactylidae. Similar, or even more elaborate, skin folds and webbing that occur in other arboreal gecko lineages are functionally associated with crypsis and parachuting locomotion^40–43^. Repeated parallel evolution of skin folds and webbing in the New Caledonia clade is similar to evolutionary patterns in Southeast Asian gekkonids, where two groups with extensive webbing and skin folds, *Ptychozoon* (gliding geckos) and *Luperosaurus* (flap-legged geckos), have been shown to be polyphyletic offshoots of the more generalized genera *Gekko* and *Lepidodactylus*^40,44,45^; the genus *Gehyra* includes similarly adapted species.

In summary, it is fitting that the evolutionary affinities of the largest gecko—*Gigarcanum delcourti*—lie with a New Caledonian clade notable for its extensive morphological and ecological diversity. Rapid evolution of phenotypic disparity is a hallmark of adaptive radiations^46^. The rapid body size evolution in the New Caledonia clade outstrips that of both Australian and New Zealand diplodactylids (^37,38^ and this study), and we document a larger frequency of transitions in tested phenotypic traits than in other diplodactylids following the mid-Cenozoic colonization of New Caledonia. This variation is especially notable because pad-bearing geckos are generally conservative in overall body shape^47^; this clearly does not preclude the evolution of variation along other morphological axes. Indeed, a tenfold difference in maximum length corresponds to a thousandfold difference in mass between *G. delcourti* and the smallest member of the New Caledonia clade, *Dierogekko baaba*, which far exceeds the degree of size disparity in the best known example of an adaptive radiation in lizards, the anoles^48^. This size likely places *G. delcourti* at the upper limit of where adhesion-based climbing is feasible^49^. Combined with the extensive variation in ecological and life history traits^9^, the New Caledonia clade as a whole represents a classic example of an adaptive radiation, with *G. delcourti* defining one extreme in form.

## Methods

### DNA isolation and generation of *G. delcourti* sequence data

We obtained DNA from the left femur of the *Gigarcanum delcourti* specimen (Fig. 1E–F). DNA isolation used reagents, tools, and laboratory facilities that had no prior contact with gecko samples. A rotary drill and grinding bit were used to produce 50 mg of bone powder from the interior of the bone shaft, to which we applied a 1/10 scale silica DNA extraction protocol^50^. 8 ng of extracted DNA was used to prepare two parallel sequencing libraries using the NEBNext Ultra II DNA Library Prep Kit for Illumina. The remaining 2 ng of isolated DNA was reserved for quality control.

The libraries were sequenced on two partial lanes of an Illumina HiSeq2000 using paired-end 100 bp sequencing. Illumina reads were cleaned and adapter-trimmed using the process_shortreads function of the Stacks software^51^. The resulting 2.28 million cleaned and trimmed reads were assembled into 860,000 contigs, which were BLAST screened to remove from downstream analysis 2478 hits to mammal, prokaryote, or other non-Sauropsida taxa flagged as likely contaminants. In addition to BLAST-based quality checks, we also PCR-amplified a portion of the 16S gene for Sanger sequencing. PCR primers are listed in Supplementary Table S1 online. Comparison of 16S sequences generated via Sanger and Illumina sequencing showed that these sequences were identical and phylogenetically nested within Diplodactylidae.

### Target locus identification and construction of comparative sequence data sets

We identified candidate loci for phylogenetic analyses by comparing contigs and individual reads against genes with available gecko sequence data. Candidate loci included those employed in previous broad gekkotan studies^7,18,19,21,29,31^ as well as 44 nuclear genes used in squamate phylogenomic studies^52^. From this comparison we identified portions of 15 focal genes suitable for use in phylogenetic analyses: 13 nuclear loci (*ACM4, CDR2, CMOS, GALR1, IFIT5, KIAA1549, KIF24, MXRA5, NOD1, PDC, PTPN12, R35, RAG1*) and two mitochondrial genes (*16S, ND2*), which together have a concatenated aligned length of 11,806 bp. The assembled *Gigarcanum delcourti* sequences included data for 12 of these genes, with the other three (*KIF24, MXRA5, PDC*) included to take advantage of dense species coverage among diplodactylids. Available sequences of these 15 genes were assembled for exemplars of 151 species of Diplodactylidae plus 16 additional gekkotan and two non-gekkotan outgroup taxa (Supplementary Table S2 online). We supplemented available data with targeted PCR and Sanger sequencing for individual frozen tissue samples of *Bavayia, Correlophus, Crenadactylus, Dactylocnemis, Dierogekko,* Diplodactylidae gen. et sp. indet., *Diplodactylus, Eurydactylodes, Hoplodactylus, Mokopirirakau, Naultinus, Oedura, Pseudothecadactylus, Rhacodactylus, Strophurus, Toropuku, Tukutuku*, and *Woodworthia*, using published or newly designed primers (Supplementary Table S1 online). Samples targeted for additional sequencing include all diplodactylid genera occurring in New Zealand and New Caledonia plus representatives of all major lineages of Australian Diplodactylidae.

One byproduct of low coverage Illumina sequencing is a surfeit of mitochondrial reads^53^ and generally the largest assembly pertains to the mitogenome. Mitogenomes for a subset of species spanning major lineages of Diplodactylidae were generated via low coverage Illumina sequencing or as by-catch from targeted enrichment sequencing for other projects. Cleaned and trimmed Illumina reads were de novo assembled using Qiagen CLC Genomics Workbench (v.12.0.2), with mitogenome fragments identified using BLAST and annotated using the MitoAnnotator web tool^54^. Gene content, order, and size were recorded for all newly assembled mitogenomes. A mitogenomic data set was assembled from these assembled sequences plus published gekkotan mitogenomes. The data set includes the *G. delcourti* mitogenome with mitogenome sequences of 27 other diplodactylids, 9 other gekkotans, and 2 non-gekkotan outgroup species, and samples major New Zealand, New Caledonian, and Australian lineages of Diplodactylidae (Supplementary Table S2 online).

### Phylogenetic analysis

The primary phylogenetic analyses used the multilocus 15-gene data set and all 169 sampled taxa. Additional analyses were performed using a 30 species subsample of the 15-gene multilocus data set, a 143 species subsample employing the 13 nuclear genes only, and the 39 species mitogenomic data set. These additional analyses minimized missing data while maintaining representative phylogenetic coverage within Diplodactylidae and were performed to ensure consistency of inferred evolutionary relationships across sampling strategies. Sequences of each locus were aligned using MAFFT v7.312^55^. Data were partitioned by gene for all phylogenetic analyses. Best-fitting models of evolution for each gene were estimated using ModelFinder implemented in IQ-TREE 2.1.2^56,57^ under the Bayesian Information Criterion (Supplementary Table S3 online).

Maximum likelihood (ML) phylogenetic analyses were performed on all four alignments using IQ-TREE 2.1.2. Branch support was assessed using 1000 rapid bootstrap replicates. We also performed separate unpartitioned single-gene ML analyses to verify that no major discordance exists in evolutionary patterns among genes. Individual gene trees were summarized in a species-tree approach using ASTRAL 5.7.1^58^. Branch support for this analysis was assessed using local quartet support values. The 169 species alignment and 30 species alignment were also used to perform ML phylogenetic analyses enforcing the following two topological constraints: constrained monophyly of *Hoplodactylus* + *Gigarcanum*, and constrained monophyly of New Zealand diplodactylids plus *Gigarcanum delcourti*. The constrained analyses used the same model parameters as the unconstrained analyses. We then performed Shimodaira-Hasegawa (SH;^59^) and Approximately Unbiased (AU;^60^) topological tests comparing unconstrained ML phylogenies to the corresponding topologically constrained phylogenies using IQ-TREE 2.1.2.

A Bayesian time-calibrated phylogeny was estimated from the concatenated 169 species data set using BEAST 2.6.3^61^. The ML tree topology estimated in IQ-TREE was used for the starting tree. The BEAST analysis was performed with a relaxed lognormal clock model and birth–death tree model. Two independent analyses were run for 100 million generations each, sampling every 10,000 generations. Tracer 1.5 was used to visualize parameters for purposes of identifying burnin and ensuring adequate chain length based on effective sample size (ESS) values > 200. The first 10 million generations of each run were discarded as burnin. A maximum clade credibility (MCC) tree was generated from the sample of credible trees. Calibration of divergence times utilized seven node constraints applied in previous studies^3,30,31^. The root prior used a normal distribution (mean = 200, sd = 20). Internal node calibrations were applied based on fossil and tectonic evidence (Supplementary Table S4 online).

### Comparative methods

We used the MCC tree estimated in BEAST to perform comparative analyses of body size evolution, external morphology, and biogeographic history within Diplodactylidae. We also performed analyses on 100 trees randomly subsampled from the posterior distribution of credible trees to account for phylogenetic uncertainty. Non-diplodactylid outgroups were pruned before performing comparative analyses to remove biases of uneven sampling. Example R scripts for each class of analysis are provided in the Supplementary Information text online.

Body size evolution was investigated using log-transformed maximum snout-vent length (SVL) data (Supplementary Table S5 online). We used the “ratebytree” function in Phytools^62^ to perform a series of censored rate tests^63,64^ to compare the rates of body size evolution between monophyletic subclades of Diplodactylidae. We compared the New Caledonian and New Zealand clades to test relative rates between the two radiations where elevated rates of body size evolution had previously been documented^37^. We also tested against evolutionary rates estimated across Diplodactylidae as a whole and performed additional analyses after pruning *Gigarcanum delcourti* from the data set to investigate how its inclusion affected estimates. Performing repeated censored rate tests on 100 trees from the BEAST posterior distribution revealed no significant variation in parameter estimates or test results. We also applied the Bayesian rate shift approach of Revell et al.^65^ to test whether a best-fitting two-rate model identifies a shift in body size evolutionary rate along the branch leading to *G. delcourti*. The analysis was performed using the evol.rate.mcmc function in phytools, run for 200,000 generations with a sampling frequency of 200. ESS values were consulted to ensure adequate chain length.

We estimated ancestral states using stochastic character mapping^66^ to investigate the evolution of toepad type, digital webbing/skin folds, and ancestral geographic regions in Diplodactylidae, with the aim of identifying the most likely character states in the common ancestor of *Gigarcanum delcourti* and its closest extant relative(s). Analyses were implemented in phytools, treating variables in each analysis as discrete characters with symmetric transition rates. Analyses using the maximum clade credibility tree were repeated 1000 times; analyses incorporating phylogenetic uncertainty using 100 trees from BEAST posterior distributions were repeated 100 times each (10,000 runs total). These recovered comparable estimates to the analyses using the MCC tree.

Geographic ranges were defined broadly as one of three states: Australia, New Caledonia (including the Loyalty Islands), or New Zealand; the geographic range of *G. delcourti* was coded as ambiguous. Discrete coding of geographic ranges excludes the possibility of reconstructing ancestral nodes as occupying multiple regions simultaneously. This approach reflects evidence indicating New Caledonian and New Zealand clades arose after dispersal from Australia rather than Gondwanan vicariance, and relatively recent ages of unambiguous diplodactylid fossils^8,34^.

Toepad and webbing/skin fold characters are based on direct observation supplemented by the literature^13^. Pad-bearing geckos are often subdivided into two general categories: those bearing typical broadened pads vs those bearing paired distal expansions (“leaf toes”; e.g.^18^). We further subdivided toepad type into the following categories (Supplementary Table S5 online): (1) broad, rounded pads with undivided lamellae; (2) broad, rounded pads with divided lamellae; (3) broad, rectangular pads with undivided lamellae; (4) narrow, rectangular pads with undivided lamellae; (5) multiple pairs of leaf-like scansors; (6) single pair of leaf-like scansors separated by claw; (7) single pair of leaf-like scansors not separated by claw; and (8) toepads absent. Webbing/skin folds are defined as (1) absent; (2) rudimentary webbing between some digits; (3) moderate digital webbing, skin folds between limbs absent; and (4) extensive webbing and/or skin folds at limb insertions.

## Supplementary Information

Supplementary Information.

Supplementary Figures.

Supplementary Tables.

## Data Availability

The new sequence data used in this study are available under GenBank accession numbers OP273955–OP274118, OP320471–OP320500 and SRA project PRJNA954403 at https://www.ncbi.nlm.nih.gov/.
