# Supplementary material for: Reappraising the evolutionary history of the largest known gecko, the presumably extinct Hoplodactylus delcourti, via high-throughput sequencing of archival DNA
Source: Sci Rep. 2023 Jun 19;13:9141. doi: 10.1038/s41598-023-35210-8 (PMC10279644; doi:10.1038/s41598-023-35210-8)
Supplement: Supplementary file 1 — Supplementary Information. [file 41598_2023_35210_MOESM1_ESM.pdf]

Supplemental Text. Example R scripts to execute each type of comparative analysis used in this study. Commands are in red.

```
##    1.Stochastic character mapping of discrete traits

##Input requires two or three files in working directory: tree ("Hoplotree.nwk" in example), character table
("Hoplodactylus_SVL.csv" in example), and if applicable, list of taxa with missing data ("missing_geog.csv" in example)

##script follows

library(phangorn)
library(phytools)
read.tree("Hoplotree.nwk")->tree     ##upload tree or trees. Replace command with read.nexus if stored in nexus format.
read.csv("Hoplodactylus_SVL.csv",row.names=1,header=TRUE)->Y   ##upload character table. Species names should match tree.
read.csv("missing_geog.csv",row.names=1,header=TRUE)->missing  ##upload list of taxa with missing data if relevant. Delete this line if no missing data.
as.character(missing$Species)->missing    ##format list of species missing characters. Delete this line if no missing data.
sapply(missing,function(x,y) which(y==x),y=tree$tip.label)->tips  ##apply missing character to tree. Delete this line if no missing data.
geographic<-as.matrix(Y)[, "Geographic"]   ##indicate which data column from table will be used in character mapping.
Geographic<-to.matrix(geographic,c("Australia","New Zealand","New Caledonia"))   ##identify character states listed in table.
rep(0.33,3)->Geographic[missing,]   ##set prior probabilities for character states.
Geographic.tree<-make.simmap(tree,Geographic,nsim=1000,message=FALSE)   ##perform stochastic character mapping.
write.simmap(Geographic.tree,append=TRUE,file="GeogMCC1000.nwk") ##export mapped trees as output file. Can re-upload later to plot or summarize.
describe.simmap(Geographic.tree)->Geographic.simm   ##summarize mapped trees for stats and plot tree.
plot(Geographic.simm,cex=0.5,fsize=0.3)   ##plot summarized tree in phylogram format. cex controls pie size, fsize controls font.
plot(Geographic.simm,cex=0.5,type="fan",ftype="off")   ##plot summarized tree in fan format. ftype="off" removes tip labels.
write.csv(Geographic.simm$ace,file="Geographic.csv")   ##export posterior probabilities for each node as table.
```

```
##     2.Censored Rate Test

##Input requires three files in working directory: two trees being compared ("NC.nwk" and "NZ.nwk" in example) and character table with continuous trait being tested ("HoploSVL.csv" in example)

##script follows

library(phytools)
first.tree<-read.tree("NC.nwk") ##loads ultrammetric tree rooted in nwk format.
first.data<-read.csv("HoploSVL.csv",row.names=1,header=TRUE,na.string=".") ##loads data table with species names in row 1. Only works if has at least two other columns of data that are numeric. Codes missing data as ".".
first.data<-first.data[first.tree$tip.label,] ## adds data as tip labels on tree. Need names to match.
first.size<-log(as.matrix(first.data)[,1]) ## log transform data if needed. Delete line if transformation isn't required. second.tree<-read.tree("NZ.nwk") ## tree to be compared to one above.
second.data<-read.csv("HoploSVL.csv",row.names=1,header=TRUE,na.string=".") ## data table.
second.data<-second.data[second.tree$tip.label,] ## labels second tree.
second.size<-log(as.matrix(second.data)[,1]) ## log transform data if needed. Delete line if transformation isn't required.
fit.size<-ratebytree(c(first.tree,second.tree),list(first.size,second.size)) ##perform test. Replace names in "list" if log transformation wasn't done.
fit.size ## view test results.
```

```
##     3.Rate Shift Test

##Input requires two files in working directory: tree ("Hoplotree.nwk" in example) and character table ("Hoplodactylus_SVL.csv" in example)

##script follows

library(phytools)
read.tree("Hoplotree.nwk" )->tree  ##loads ultrametric tree rooted in nwk format.
read.csv("Hoplodactylus_SVL.csv",row.names=1,header=TRUE)->testdata  ##loads character table of continuous trait.
as.vector(test$logSVL)->values ##pulls trait values from logSVL column of table.
names(values)<-row.names(testdata)  ##associates taxon names with trait values.
values ##check that previous operation worked.
evol.rate.mcmc(tree, values, ngen=200000, control=list(sample=200))->rate ##performs rate shift test.
rate  ##check that results are stored in this object.
write.csv(rate$mcmc,"mcmcfile.txt")  ##writes MCMC results to file that can be checked for convergence using Tracer or another program.
summary(rate)->obj  ##summarizes MCMC results.
obj  ##check that summary data are stored.
plot(obj,method="edge.prob",piecol=c("blue","lightgrey"), ftype="off",mar=c(0.1,0.1,5.1,0.1)) ##plots summarized frequency of inferring rate shift along each branch.
```
