# Supplementary material for: Reappraising the evolutionary history of the largest known gecko, the presumably extinct Hoplodactylus delcourti, via high-throughput sequencing of archival DNA
Source: Sci Rep. 2023 Jun 19;13:9141. doi: 10.1038/s41598-023-35210-8 (PMC10279644; doi:10.1038/s41598-023-35210-8)

Figure S1. Maximum likelihood phylogeny using the 169 species multilocus data set. Bootstrap support values are indicated at nodes.

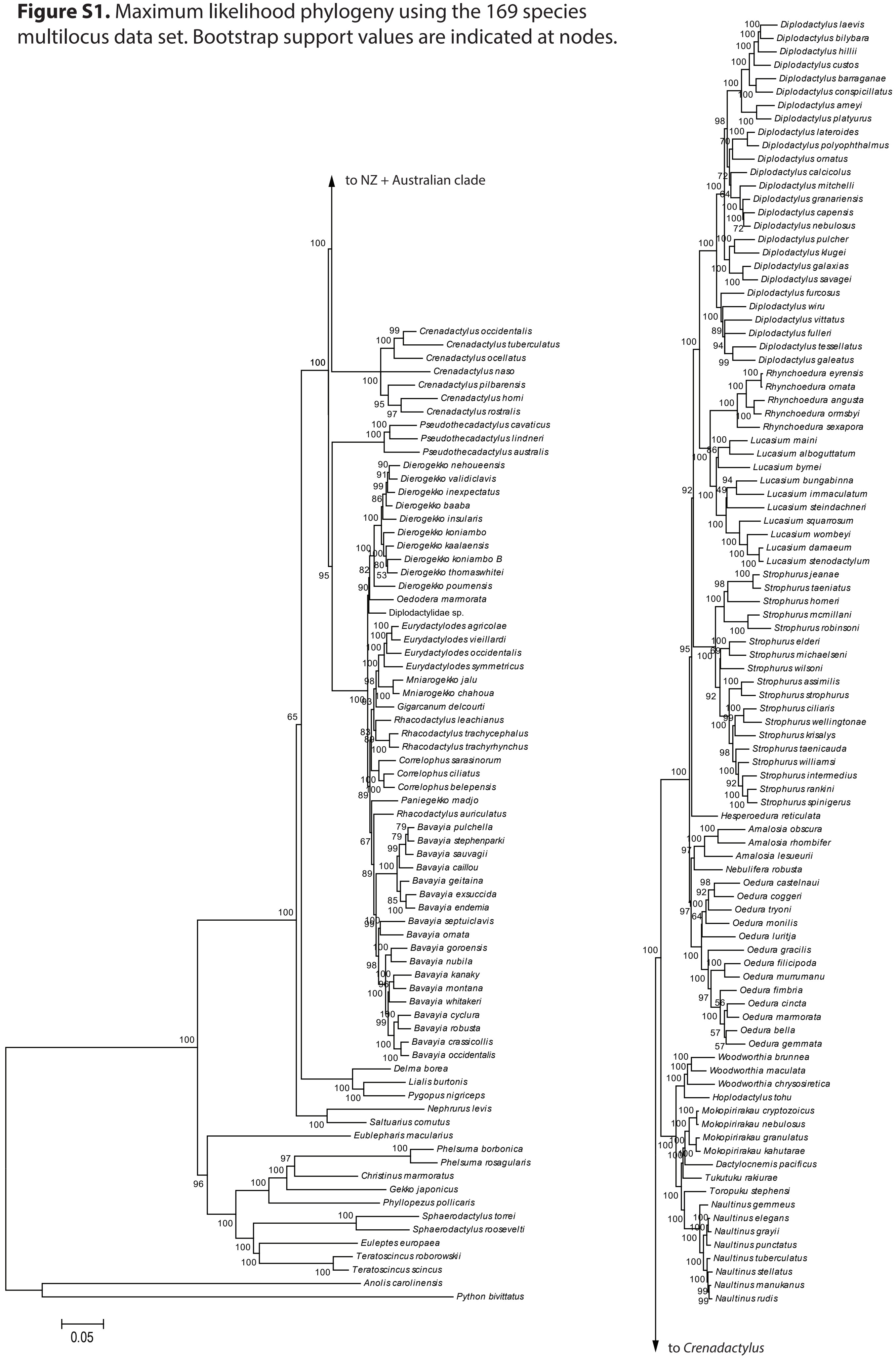

Figure S2. Maximum likelihood phylogeny using the 39 species mitogenome data set. Bootstrap support values are indicated at nodes.

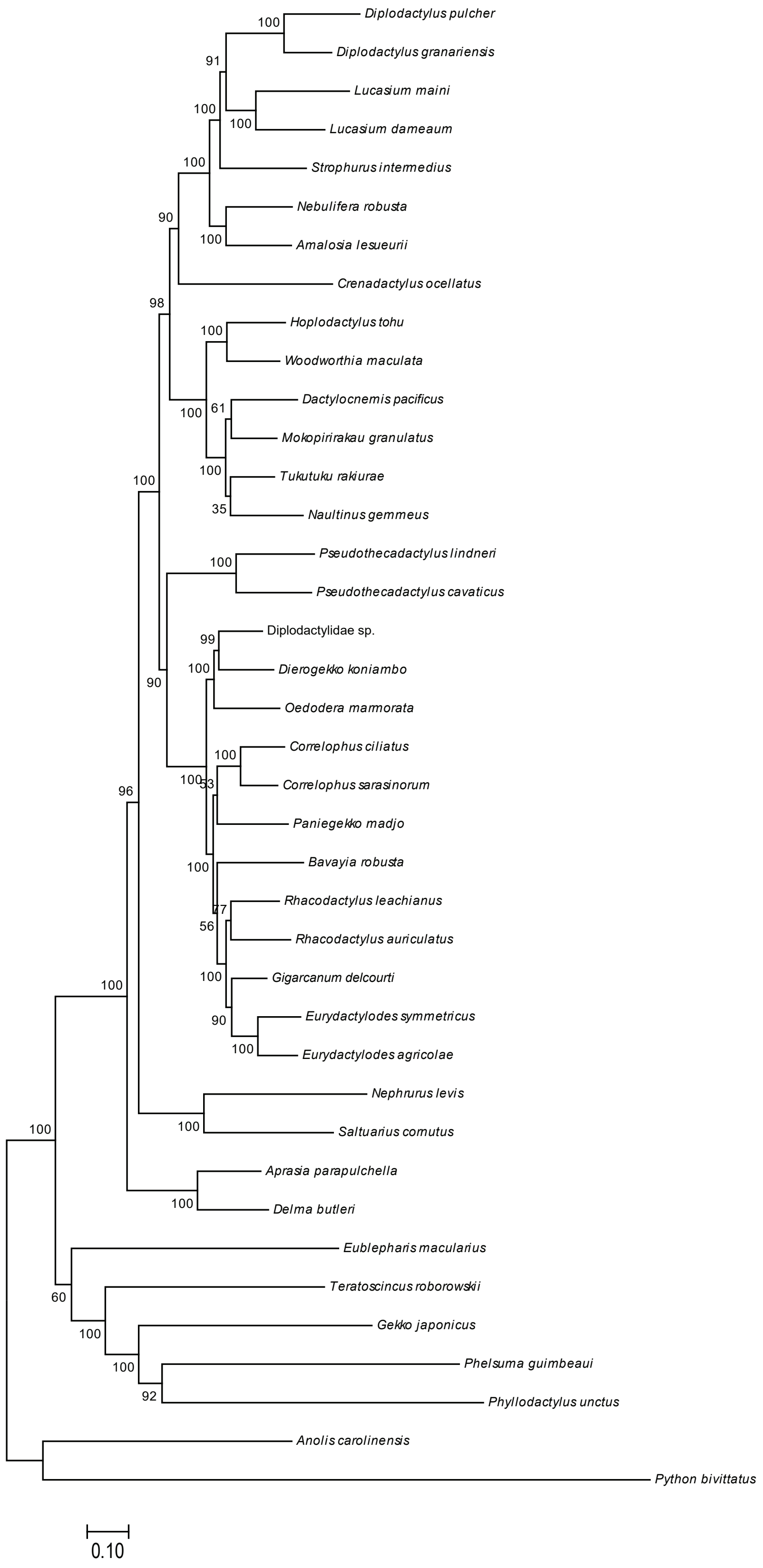

Figure S3. Maximum likelihood phylogeny using the 30 species multilocus data set. Bootstrap support values are indicated at nodes.

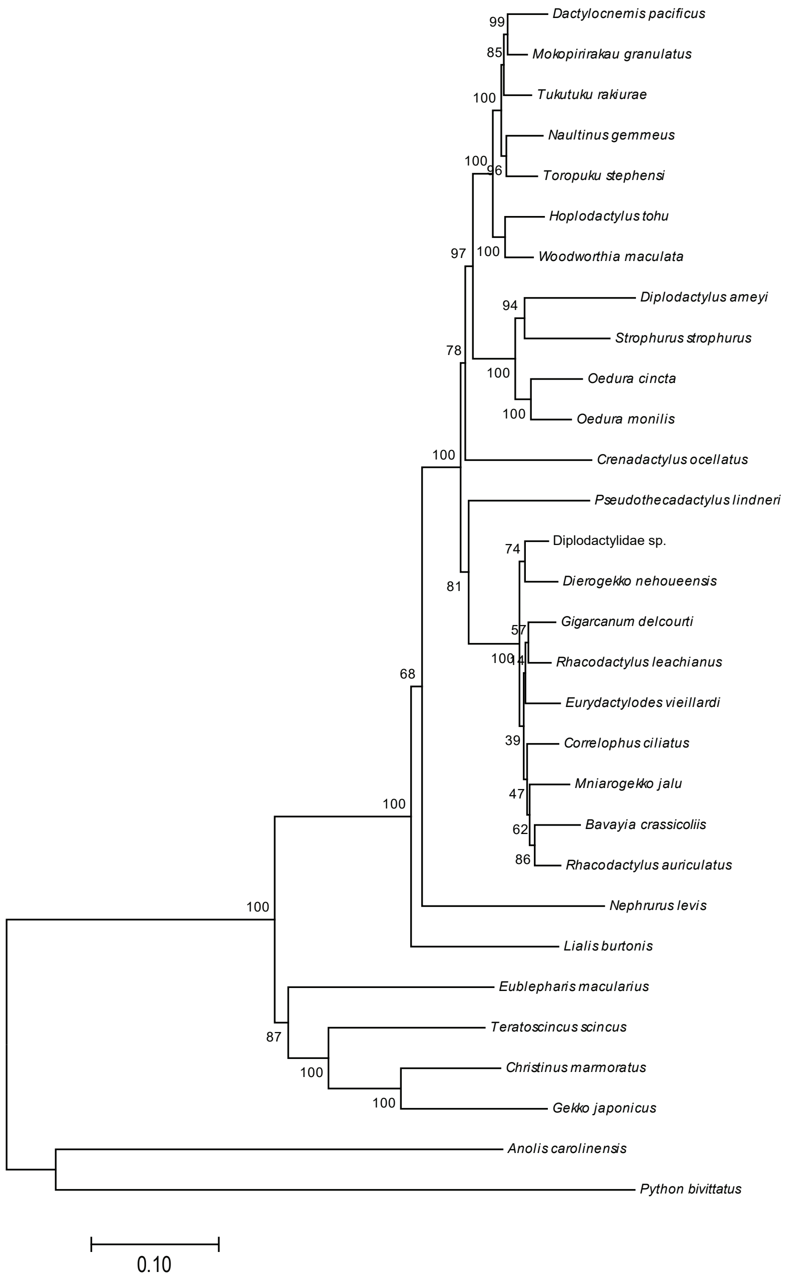

Figure S4. Maximum likelihood phylogeny using the 13 nuclear loci from the multilocus data set (143 sp.). Bootstrap support values are indicated at nodes.

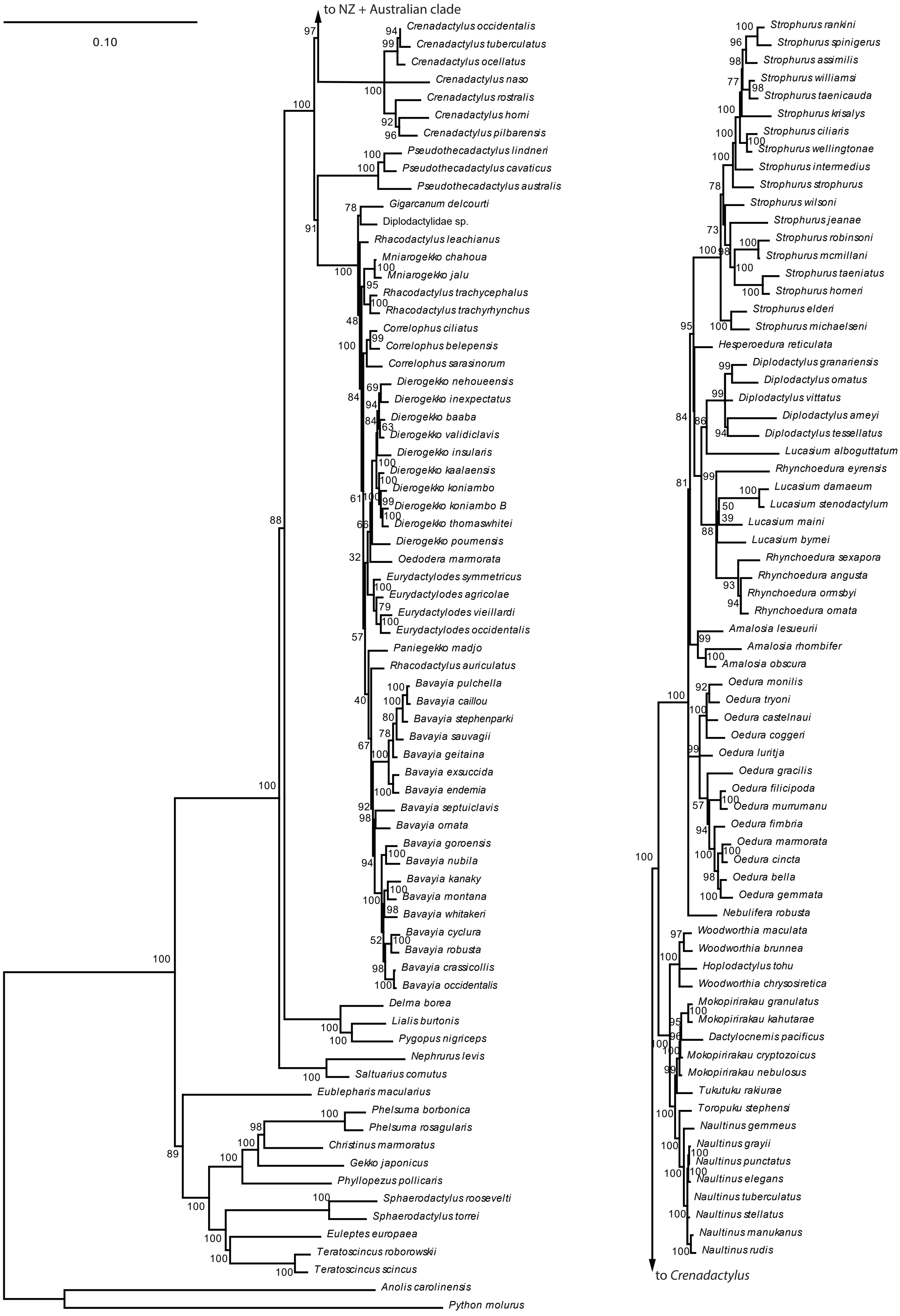

Figure S5. ASTRAL species tree using the 169 species multilocus data set. Local quartet support values are indicated at nodes. Note that ASTRAL does not estimate the lengths of terminal branches; these are depicted with dashed lines.

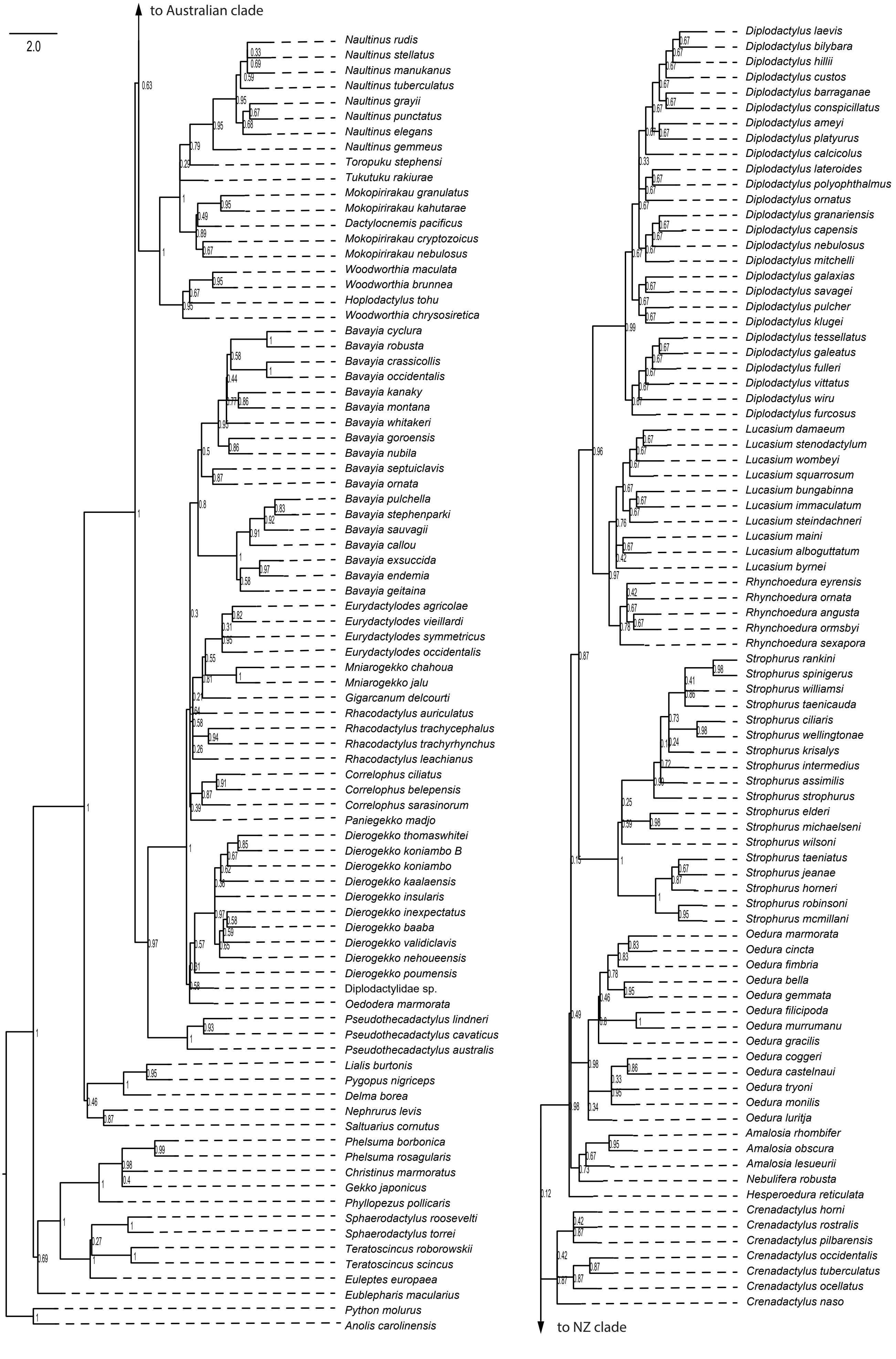

Supplement: Supplementary file 2 — Supplementary Figures. [file 41598_2023_35210_MOESM2_ESM.pdf]
